# Expectant Treatment in Caries of the Ankle in Children

**Published:** 1880-06

**Authors:** 


### EXPECTANT TREATMENT IN CARIES OF THE ANKLE IN CHILDREN.

Dr. Gibney, of the Hospital for the Ruptured and Crippled, New York, from an analysis of thirty cases of this disease, draws the following conclusions:

1. Many children annually undergo amputation of the foot for caries of the ankle, when by conservatism and a proper amount of respect for the vis medicatrix naturæ, the member could be saved, the child be spared the mortification of being thus hopelessly maimed, and surgery itself be ennobled.

2. Excision as a rule is not attended with as good results in children as authorities have led us to expect, and is *rarely ever justifiable.*

3. Partial excisions, the passage of tents through the joints, and other operative procedures offer no advantages over the expectant plan.

4. Nature herself, unaided by art, gets useful limbs, but as a rule, anchylosis varying in degree and deformity more or less marked.

5. The expectant plan of treatment, fully carried out, assures us of more results that are perfect, and more limbs that are useful without the aid of support, than does any other plan known to the profession.—*American Journal Obstetrics, April, 1880.*

